# Options to Expand HIV Viral Load Testing in South Africa: Evaluation of the GeneXpert® HIV-1 Viral Load Assay

**DOI:** 10.1371/journal.pone.0168244

**Published:** 2016-12-16

**Authors:** Natasha Gous, Lesley Scott, Leigh Berrie, Wendy Stevens

**Affiliations:** 1 Department of Haematology and Molecular Medicine, School of Pathology, Faculty of Health Sciences, University of Witwatersrand, Johannesburg, South Africa; 2 National Priority Program of the National Health Laboratory Services, Johannesburg, South Africa; Centers for Disease Control and Prevention, UNITED STATES

## Abstract

**Background:**

Expansion of HIV viral load (VL) testing services are required to meet increased targets for monitoring patients on antiretroviral treatment. South Africa currently tests >4million VLs per annum in 16 highly centralised, automated high-throughput laboratories. The Xpert HIV-1 VL assay (Cepheid) was evaluated against in-country predicates, the Roche Cobas Taqmanv2 and Abbott HIV-1RT, to investigate options for expanding VL testing using GeneXpert’s random access, polyvalent capabilities and already established footprint in South Africa with the Xpert MTB/RIF assay (207 sites). Additionally, the performance of Xpert HIV-1VL on alternative, off-label specimen types, Dried Blood Spots (DBS) and whole blood, was investigated.

**Method:**

Precision, accuracy (agreement) and clinical misclassification (1000cp/ml) of Xpert HIV-1VL plasma was compared to Taqmanv2 (n = 155) and Abbott HIV-1 RT (n = 145). Misclassification of Xpert HIV-1VL was further tested on DBS (n = 145) and whole blood (n = 147).

**Results:**

Xpert HIV-1VL demonstrated 100% concordance with predicate platforms on a standardised frozen, plasma panel (n = 42) and low overall percentage similarity CV of 1.5% and 0.9% compared to Taqmanv2 and Abbott HIV-1 RT, respectively. On paired plasma clinical specimens, Xpert HIV-1VL had low bias (SD 0.32–0.37logcp/ml) and 3% misclassification at the 1000cp/ml threshold compared to Taqmanv2 (fresh) and Abbott HIV-1 RT (frozen), respectively. Xpert HIV-1VL on whole blood and DBS increased misclassification (upward) by up to 14% with increased invalid rate. All specimen testing was easy to perform and compatible with concurrent Xpert MTB/RIF Tuberculosis testing on the same instrument.

**Conclusion:**

The Xpert HIV-1VL on plasma can be used interchangeably with existing predicate platforms in South Africa. Whole blood and DBS testing requires further investigation, but polyvalency of the GeneXpert offers a solution to extending VL testing services.

## Introduction

HIV viral load (VL) testing is used to monitor the effectiveness of antiretroviral therapy (ART) after treatment initiation, identify early virological failures to target adherence counselling and guide treatment switch [1]. South Africa has used VL testing preferentially for monitoring of ART since program inception, which, is now also strongly recommended by the World Health Organisation (WHO) [2]. There are currently over 3900 National Department of Health (NDoH) clinics providing ART initiation and monitoring services in South Africa [3]. All VL testing requirements to service these clinics is performed and managed by the National Health Laboratory service (NHLS) with oversight from the National Priority Programme (NPP), through 16 centralised VL laboratories across the 9 provinces. These services manage over four million VL tests annually and rely on high throughput plasma-based platforms, namely the Roche Cobas® Taqman/Cobas® Ampliprep version 2 (Taqman v2), Roche Cobas® 6800/8800 (Roche Molecular Diagnostics, Branchburg, US) and Abbott HIV-1 RealTi*m*e (Abbott HIV-1 RT) (Abbott Molecular, Des Plaines, Illinois).

The VL testing volumes in South Africa are expected to rise significantly to meet the monitoring needs of the UNAIDS ‘90-90-90’ HIV treatment targets, which aim to place 90% of those eligible for ART on treatment and ensure 90% are also virally suppressed by 2020 [4]. To ensure that VL testing volume requirements are met and are within a clinically relevant turn-around time, a hybrid testing model consisting of both laboratory and point of care testing (POCT), as recently proposed for CD4 testing services [3], may be required. Adapting this model for VL testing could require the expansion of existing centralised, high-throughput VL testing capacity, dried blood spot (DBS) specimen transport and testing and extension of VL testing services to lower throughput decentralised sites.

The GeneXpert® (GX) technology has polyvalent (multi-test) capabilities and an already established footprint in South Africa for tuberculosis (TB) diagnosis (207 smear microscopy centers across 9 provinces). The GeneXpert platform is modular and caters to a range of testing needs from high (GX 48–80 module instruments) and medium throughput (GX 4–16 module instruments) to low throughput with the soon to be introduced POC Xpert Omni platform (single module) [5]. We therefore, investigated the performance of the Xpert HIV-1 VL assay as an option to extend VL testing services in South Africa.

The Xpert® HIV-1 VL assay received CE-IVD status in 2014 [6] and is able to detect HIV-1 RNA in groups M, N and O to provide a quantitative VL result on plasma in 92 minutes (<2 minutes hands-on time and 90 minutes testing time), with a lower limit of quantification of 40copies/ml (cp/ml) [7]. Evaluation studies to date have reported good performance of the Xpert HIV-1 VL assay against existing technologies such as the Abbott HIV-1 RT and Aptima HIV Dx (Hologics®, Inc) on plasma [8–10] and therefore, warranted evaluation in the South African setting to consider criteria for implementation. This evaluation also included developing protocols for DBS and whole blood specimen types (for which the manufacturer has no claim), as well as development of a quality monitoring program to support potential VL implementation for all specimen types.

## Method

### Study site description

Ethics was approved by the University of the Witwatersrand Human Research Ethics Committee (M110139) to obtain four 5ml tubes of anti-coagulated (EDTA.K_3_), venous-derived whole blood from adult ART patients. Written informed consent was obtained when patients presented at the phlebotomy room of Themba Lethu HIV Clinic, Helen Joseph Hospital, Johannesburg, for routine VL monitoring. Following routine blood draw, an additional four EDTA.K_3_ blood tubes were obtained and couriered the same day (approximately 30 minutes) to the University of the Witwatersrand Diagnostics Research testing laboratory.

### Specimen types

To verify the Xpert HIV-1 VL assay (referred to as Xpert VL) as ‘fit-for-purpose’ prior to clinical specimen testing, a standardised, 42 member, frozen, subtype-C plasma panel (termed the South African Viral Quality Assessment–SAVQA) [11], was included in the evaluation. The SAVQA panel consisted of 22 HIV-negative specimens and 20 HIV-positive specimens ranging from 750 to 21000cp/ml (2-4log cp/ml) and is designed to measure accuracy and precision.

**Fig 1:**
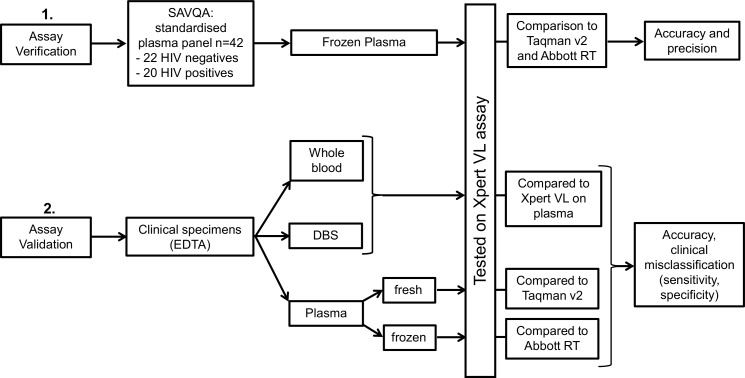
Validation plan for the Xpert VL assay. The three specimen types used for validating the Xpert VL assay and statistical methodology employed.

For assay validation, three clinical specimen types were tested on the Xpert VL assay (Fig 1); whole blood, plasma and DBS. DBS were prepared immediately upon specimen receipt, by pipetting 70μl of whole blood per spot onto a Munktell 903 filter card (LabMate Pty, Ltd, Cape Town, South Africa) (four spots per card), air dried for 2 hours and then stored at -20°C in a zip-lock plastic bag with dessicant for later testing. Whole blood did not require any pre-processing before testing on the Xpert VL assay. To obtain plasma, residual whole blood was centrifuged at 3000rpm for 15 minutes using a Hettich EBA-20 centrifuge (Hettich AG, Germany).

### Xpert HIV-1 VL testing procedure for different specimen types

Three GXIV (four module) instruments were available for the study. Whole blood Xpert testing was performed on the same day as venepuncture. Testing involved the addition of 1.2ml of a guanidinium thiocyanate-based buffer (proprietary buffer, not supplied with Xpert kit) to 100μl of whole blood in a microcentrifuge tube. The tube was inverted several times to mix and 1.2ml of the blood/buffer mixture was transferred by volumetric pipette directly into the Xpert VL cartridge (1.2ml was added to standardise and ensure no volume related errors).

Plasma testing was performed within a 24-hour period of specimen receipt and involved addition of 1.2ml of plasma directly into the Xpert VL cartridge. Any residual plasma specimens were stored at -70°C.

DBS were tested within a month of specimen receipt by placing a single DBS into a 15ml nunc tube, adding 1.3ml of the guanidinium thiocyanate-based buffer (same as used for whole blood) and incubating in a thermomixer at 56°C, 500rpm, for 15 minutes (Eppendorf AG, Hamburg, Germany). A volume of 1.2ml of the lysate was added directly into the Xpert VL cartridge.

The Xpert VL assay integrates automated sample purification, reverse transcriptase amplification and real-time detection (RT-PCR) of the 5’-LTR region of HIV-1, in a closed cartridge system. Within the cartridge, two controls, a high and low, are provided for quantitation of HIV-1 RNA and to ensure adequate sample processing and amplification [7]. A probe check control (PCC) is present to verify reagent rehydration, PCR tube filling in the cartridge, probe integrity, and dye stability. Currently, a quantifiable result is available in 90 minutes from cartridge loading. Any values reported by Xpert VL as <40cp/ml (lower limit of detection) were reported as 39cp/ml for the purposes of quantitative statistical data analysis only.

### Statistical methodology

The statistical methodology employed for specimen types is detailed in Fig 1.

Assay Verification: Results of the Xpert VL assay on the SAVQA panel were compared to published performance criteria [11, 12] using the Taqman v2 and Abbott HIV-1 RT platforms.

Assay validation on clinical specimens: Xpert VL assay precision (variability), both intra (within module) and inter (between modules), was determined using three VL log categories (log 2.0cp/ml, log 3.0cp/ml and log 4.0cp/ml) of fresh clinical plasma specimens. Intra-variability was calculated using the mean and standard deviation (SD) of five replicate specimens tested on the same module within the same day. Inter-variability was calculated from testing the same plasma specimen across all four modules of a GXIV platform.

Agreement (accuracy) was determined between the Xpert VL assay and the Taqman v2 (standard of care/SOC) on patients’ paired clinical plasma specimens for the period November 2014 to July 2015. Any residually stored plasma specimens were also tested on the Abbott HIV-1 RT assay as an added comparator to the Xpert VL assay. The lower limit of detection for Taqman v2 is cited as 20cp/ml by the manufacturer (reported as 19cp/ml for quantitative analysis) and for Abbott HIV-1 RT is 40cp/ml (reported as 39cp/ml for quantitative analysis). Agreement was measured by concordance correlation [13] and the Bland-Altman method [14] (quantified specimens only). Concordance correlation (*pc*) was calculated as a measure of agreement strength (accuracy and precision) between the Xpert VL assay and described reference methods [13, 15]. A Passing and Bablok regression was also plotted to indicate the measure of bias and precision [16]. Overall agreement between the data pairs is represented as a percentage similarity CV (percentage similarity standard deviation/ percentage similarity mean) [17].

The sensitivity, specificity, positive predictive value (PPV) and negative predictive value (NPV) (including 95% confidence intervals), as well as possible misclassification of the Xpert VL on plasma at the 1000cp/ml (log 3.0) clinical treatment failure threshold was determined using Taqman v2 and Abbott HIV-1 RT as the reference standards. Misclassification refers to specimens either incorrectly resulted as below the 1000cp/ml threshold (downward misclassified/incorrectly identified as virological success) or above the threshold (upward misclassified/incorrectly identified as virological failure) compared to reference technology. The sensitivity, specificity and misclassification of the Xpert VL on whole blood and DBS were also determined at the 1000cp/ml threshold, using plasma Xpert VL results as the reference standard. All statistical analyses were performed using excel, STATA version 12, MedCalc and medical calculator [18]. Qualitative variables such as errors and invalids, as well as ease of use, were reported for future implementation considerations.

### Development of a VL quality monitoring program

**Fig 2:**
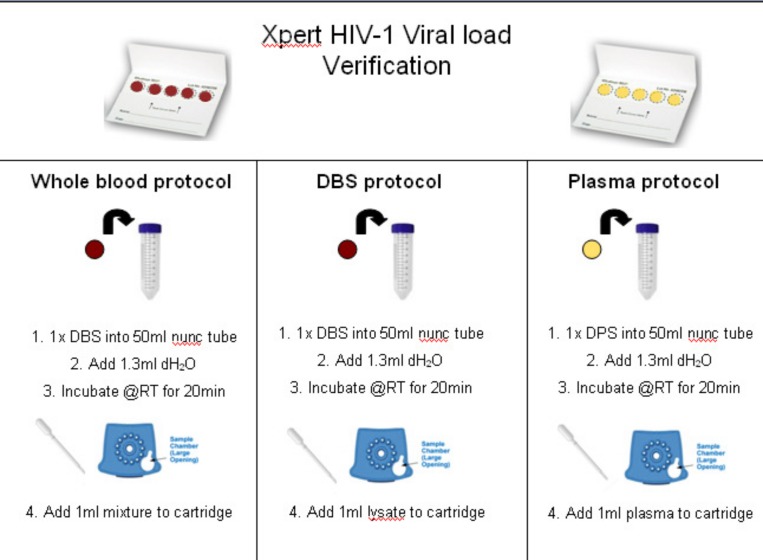
Protocol for testing of the DPS and DBS on the Xpert VL assay. Schematic of the use of dried viral spots (DVS) for quality management of the GeneXpert instrument with the Xpert HIV-1 VL assay.

A quality monitoring program was developed for verifying new instruments, training operators and potentially as an external quality assessment (EQA) matrix specifically for the Xpert VL assay, as was developed by this group for the Xpert MTB/RIF program [19, 20]. Dried plasma spots (DPS) were prepared using plasma containing a high VL (log 5) obtained from the SANBS (South African National Blood Service). A total of 70μl of plasma was added directly onto Munktell 903 filter cards (LabMate, SA) and allowed to air-dry for 2 hours before placing in a sealable plastic bag with desiccant. Dried blood spots (DBS) were prepared by diluting the same plasma in a 1:1 ratio with stabilised blood material (cd Chex, Streck, Inc, Omaha, NE) and spotting 70μl onto the Munktell 903 filter cards. One DPS and one DBS were tested at baseline (day of preparation) on the Xpert VL assay and the remaining spots were stored at room temperature and -20°C for stability testing over 42 days. For testing on the Xpert VL assay, a single 70μl spot was resuspended in 1.3ml distilled water as per Fig 2, incubated for 20 minutes at room temperature with intermittent mixing, and the re-suspended liquid tested using the Xpert VL cartridge as a clinical specimen.

## Results

### Xpert VL assay verification

Table 1 outlines the performance in terms of assay precision and accuracy, of the Xpert VL assay and the two comparator reference technologies (Roche and Abbott) on the frozen, subtype C SAVQA plasma panel. Apart from one error result reported on Xpert VL on an HIV-negative specimen (instrument error), all remaining specimens (n = 21) showed 100% result concordance with reference technology. The Xpert VL had good intra-variability (precision) (SD = 0.16logcp/ml) compared to both reference methods across the tested range of the SAVQA panel and within the categories 2-4logcp/ml. Xpert VL showed a negative bias compared to Taqman v2 (generating overall lower values than Taqman v2), but a positive bias compared to Abbott HIV-1 RT (generating higher values than Abbott HIV-1 RT). Xpert VL has overall good agreement (%CV) with both reference methods on subtype C plasma.

**Table 1:** Method comparison for the evaluation of Xpert VL compared to Taqman v2 and Abbott HIV-1 RT on the frozen SAVQA panel.

**Assay precision (variability) on SAVQA frozen plasma panel**					
	**Precision**				
	**Intra-variability (log cp/ml)**			**Inter-variability (log cp/ml)**	
Xpert VL, n = 20, SD	0.16log cp/ml			n/a	
Taqman v2, n = 20, SD	0.15log cp/ml			n/a	
Abbott HIV-1 RT, n = 20, SD	0.15log cp/ml			n/a	
Assay accuracy* (bias and agreement) on SAVQA frozen plasma panel					
**Xpert VL versus**		**Log bias (absolute difference), SD**	**Percentage similarity mean, SD, (CV%)**		***pc* (95%CI)**
Taqman v2, n = 20^a^		-0.18cp/ml,0.1cp/ml	99, 1.5 (1.5%)		0.922(0.82, 0.97)
Abbott HIV-1 RT, n = 20^a^		0.06cp/ml,0.08cp/ml	102, 0.9 (0.9%)		0.918(0.84, 0.96)

^a^quantifiable specimens only

### Performance of the Xpert VL assay on clinical plasma specimens

There were 158 participants enrolled in the study; 53.8% were female (85/158) with a median age of 42 years [IQR 36–50 years]. VL results could not be reported on three SOC specimens (n = 2 sample clotting, n = 1 invalid) tested by Taqman v2 (n = 155 results available for analysis). Five specimens reported an error on the Xpert VL assay; however, all five could generate results on repeat testing. Residual plasma (tested frozen in batches) from 145 specimens was available for Abbott testing and no errors or invalids were reported on this platform.

**Fig 3:**
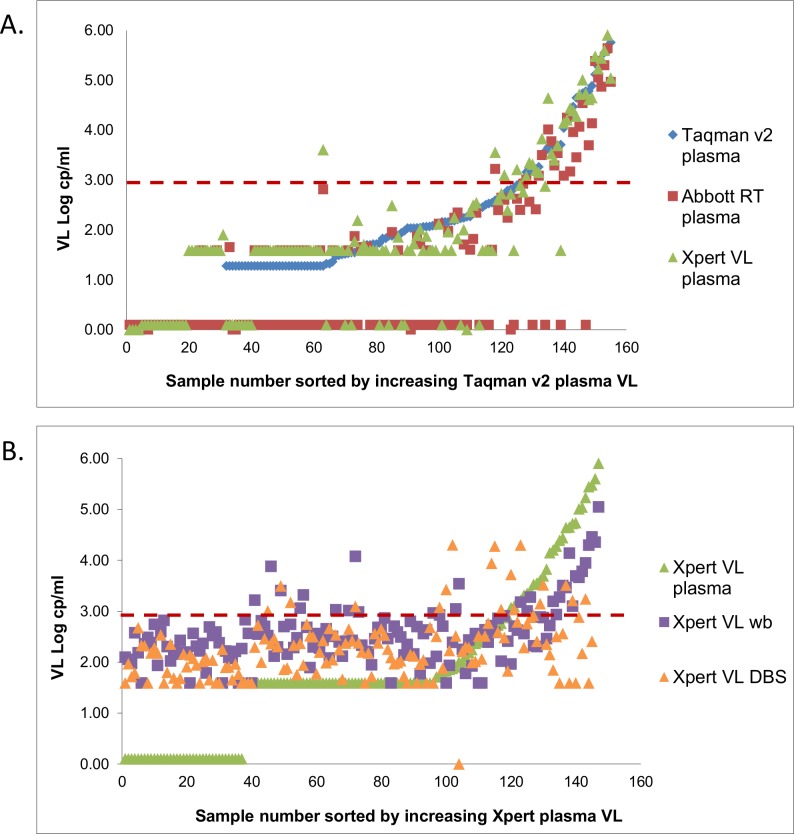
Scatter plots showing comparison of log VL values for Taqman v2, Abbott HIV-1 RT and Xpert VL assay. (A) Xpert HIV-1 (green) compared to Taqman v2 (blue) and Abbott HIV-1 RT (red) on plasma specimens. The vertical axis represents the log VL and the horizontal axis represents the sample number sorted by the Taqman v2 assay; B) Xpert HIV-1 plasma (blue) compared to Xpert HIV-1 whole blood (red) and Xpert HIV-1 DBS (green). The vertical axis respresents the log VL and the horizontal axis is sample number sorted by Xpert HIV-1 plasma VL values. The red dotted line in both plots highlights the 1000cp/ml clinical threshold.

Fig 3A visually represents the results from all three VL assays, and highlights the manufacturer’s lower limit of detection for the Taqman v2 (20cp/ml) compared to 40cp/ml for the Abbott HIV-1 RT and Xpert VL assays. Taqman v2 generated quantifiable results on 25% more specimens than the Abbott HIV-1 RT and Xpert VL. Also noted, is the increase in number of specimens that were reported on Xpert VL as <40 cp/ml (detected but not quantifiable) (39%) compared to only 10% with similar reporting on the Abbott HIV-1 RT.

Xpert VL assay intra-precision was acceptable (≥0.19 SD)[11] at all log categories tested but showed marginally higher inter-variability at the lower log2 category (Table 2).

**Table 2:** Xpert VL assay precision and method comparison on clinical plasma specimens.

**Xpert VL assay precision on fresh clinical plasma specimens**				
**Log category tested**	**Intra-variability, mean (SD) log cp/ml**		**Inter-variability, mean (SD) log cp/ml**	
log 2cp/ml	2.72 (0.03)		2.2 (0.51)	
log 3cp/ml	3.38 (0.12)		3.2 (0.1)	
log 4cp/ml	3.84 (0.13)		4.6 (0.1)	
Xpert VL assay accuracy* (bias and agreement) on clinical plasma specimens				
**Xpert VL versus**	**Log bias (absolute difference), SD**	**Percentage similarity mean, SD, (CV%)**		***Pc* (95%CI)**
Taqman v2 (fresh),n = 53^a^	0.03 (0.32) cp/ml	101, 5.9 (5.9)		0.96 (0.94, 0.98)
Abbott HIV-1 RT(frozen), n = 45^a^	0.34 (0.37) cp/ml	106, 7.1 (6.68)		0.91 (0.85, 0.95)

^a^quantifiable specimens only

**Fig 4:**
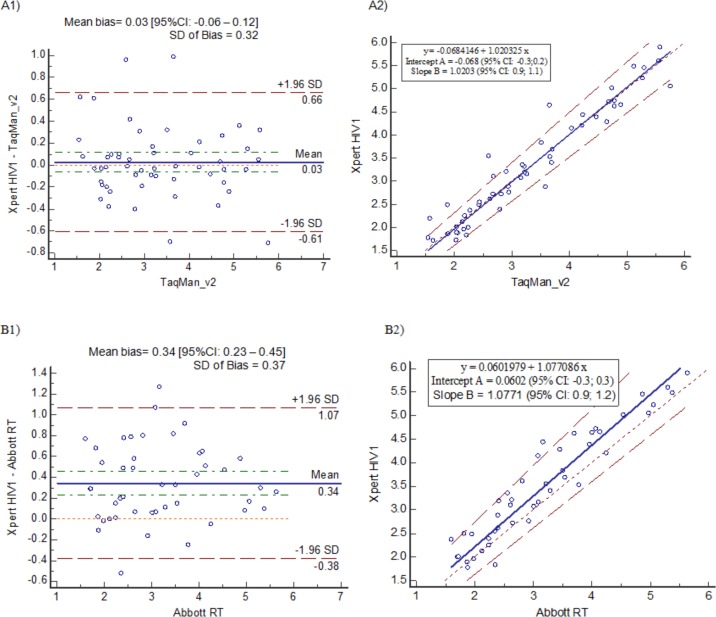
Bland-Altman and Passing and Bablok regression scatter plots. Xpert HIV-1 VL versus (A) Taqman v2 (n = 53) and (B) Abbott HIV-1 RT (n = 45); (1) Bland Altman difference plots show the mean bias (solid line) and confidence intervals (dashed line); (2) Passing and Bablok regression analysis show the regression line (solid) with confidence intervals (dashed). Legends highlight the bias and regression equation.

Assay accuracy (Table 2) among paired clinical plasma specimens was also acceptable compared to both Taqman v2 (fresh plasma) and Abbott HIV-1RT (frozen plasma), with acceptable standard deviation of the bias on quantified specimens (~0.3log cp/ml), although the concordance correlation was higher with Xpert VL compared to Taqman v2 than with Xpert VL compared to Abbott HIV-1 RT. This is further visualised on the Bland-Altman and Passing and Bablok regressions (in Fig 4), which show less scatter between Xpert VL and Taqman v2 (A1 and A2) than between Xpert VL and Abbott HIV-1 RT (B1 and B2).

Overall, the good agreement between Xpert VL and both reference assays on plasma specimens resulted in little misclassification at the 1000cp/ml clinical threshold (Table 3), with good sensitivity and specificity. Only 2/5 Taqman v2 results (3762cp/ml and 5090cp/ml) were downward misclassified by Xpert VL (as 752cp/ml and <40cp/ml respectively). This translated into a PPV of 86.7% [CI: 69.3; 96.2] and NPV of 98.4% [CI: 94.3; 99.8] for the comparison against Taqman v2. Five specimens were upward misclassified by Xpert VL against Abbott HIV-1 RT, resulting in a PPV of 83.9% [CI: 66.3; 94.5] and NPV of 100% [CI: 96.9; 100].

**Table 3:** Clinical misclassification of the Xpert VL assay tested on plasma, whole blood and DBS specimens.

Clinical misclassification at 1000cp/ml threshold			
Xpert VL on plasma versus	Total Misclassi-fication, n (%)	Sensitivity, %(95% CI)	Specificity, % (95% CI)
Taqman v2 plasma n = 155	5/155 (3.2)	92.9 (77, 99)	96.9 (92, 99)
Abbott HIV-1 RT plasma n = 145	5/145 (3.4)	100 (85, 100)	95.9 (91, 99)
Xpert VL on whole blood n = 147	21/147 (14.3)	60.7 (41, 79)	91.6 (85, 96)
Xpert VL on DBS n = 145	18/145 (12.5)	50 (31, 69)	96.6 (92, 99)

### Performance of the Xpert VL assay on clinical whole blood and DBS specimens

Fig 3B shows the performance of the Xpert VL on whole blood and DBS specimens. No specimens reported as HIV-positive on plasma were reported as ‘lower than detectable limit’ when tested on either whole blood or DBS. Compared to Xpert VL on plasma, whole blood and DBS generated quantifiable results in 60% and 52% more HIV-positive specimens, respectively. This resulted in up to 14% more specimens misclassified at 1000cp/ml (Table 3), with all generating higher values for whole blood (bias -0.47log cp/ml [SD 0.77cp/ml]) and DBS (bias -0.76log cp/ml [SD 0.78cp/ml]) than plasma. Overall, both specimen types generated acceptable specificity similar to plasma testing (Table 3).

### Considerations for implementing Xpert HIV-1 VL into routine practise

Xpert VL testing generated similar error rates across all specimen types: 3.1% (5/158) for plasma, 2.5% (4/158) for whole blood and 4.6% (7/150) for DBS. All errors (5006/5007) reported were probe check failures indicating internal cartridge problems due to probes not being reconstituted properly, incorrect reaction tube filling or probe degradation (7). Plasma was the only specimen type that could be repeated on residual specimen without further reporting issues. Plasma specimens did not generate any invalids, however, a high invalid rate was observed for whole blood (19%; 30/158) and DBS (18%; 27/150), indicating internal control failure.

**Fig 5:**
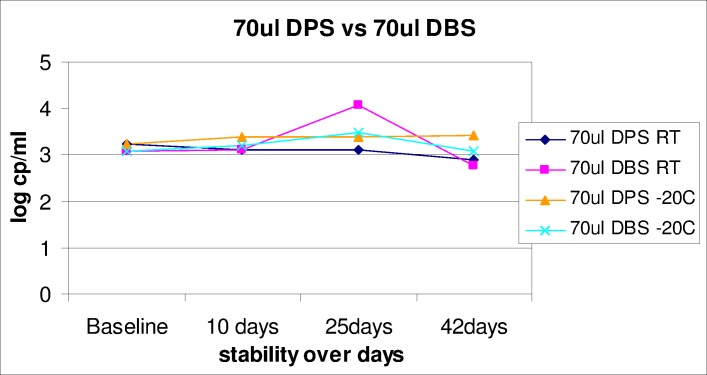
A line plot showing the stability of dried viral spots (DVS) for instrument and assay verification. The log VL reported on Xpert HIV-1 was measured on DVS stored at room temperature and -20C over 42 days.

Quality: The dried viral spots (DPS and DBS) were easy to prepare and compatible with the Xpert VL testing protocols, and appeared suitable for instrument verification for the Xpert VL assay. The DPS and DBS remained stable after 42 days of storage at either room temperature or -20°C, with no degradation of virus on the filter cards as evident in Fig 5.

## Discussion

Expanding HIV VL testing services is challenging as logistical complexities related to sample transport, the need for infrastructure, complex equipment, training, cold chain and instrument maintenance, exist. The modular, random access design of the GeneXpert technology makes this platform attractive to diagnostic programs seeking a total coverage solution. This platform can span diagnostic service provision at decentralised (remote) settings using single module instrument formats (and soon the Xpert Omni POC format) to highly centralised testing sites. The polyvalent capabilities of the GeneXpert further expand the diagnostic testing options, and thus we investigated the Xpert VL assay as an option to extend VL services using an existing GeneXpert technology footprint in South Africa.

The Xpert HIV VL assay showed comparable performance to existing high throughput, predicate technologies (Taqman v2 and Abbott HIV-1 RT) on a standardized, frozen plasma panel (HIV-subtype C) as well as fresh and frozen clinical plasma specimens from a Johannesburg HIV ART treatment clinic. The Xpert VL showed good precision on plasma specimens across the VL ranges tested (log2.0–4.0cp/ml), acceptable bias and good concordance correlation with reference technologies. It is well described that Taqman v2 has a lower limit of detection than Abbott HIV-1 RT (20 versus 40cp/ml) and Taqman v2 generates higher values than Abbott HIV-1 RT. In our study, the Xpert VL tended to generate lower values than Taqman v2 and higher values than Abbott HIV-1 RT. The comparison to Abbott HIV-1 RT was however, performed on residual, frozen plasma specimens and even though the performance was acceptable the freeze/thaw cycle may have impacted on the accuracy of these results. Regardless, the Xpert VL technology proved to be a suitable replacement/extension to either of these technologies within the South African VL testing program without the need to anticipate clinically relevant bias. This was further emphasized by the low level of misclassification shown by the Xpert VL at the 1000cp/ml clinical threshold compared to either of the reference technologies. The NPV (98.4–100%) confirms that an Xpert VL result below the 1000cp/ml threshold reports true virological suppression and hence, this assay may be used for HIV VL treatment monitoring in South Africa.

The results of the Xpert VL assay on whole blood and DBS for VL monitoring appeared promising and use of these specimen types on the GeneXpert platform could further extend the potential implementation options for this technology. Although these specimen formats increased the misclassification rates by up to 14% (upward misclassification) compared to their plasma results, specificity was still higher than four out of five technologies mentioned by WHO [21]. In clinical terms, this would translate into more patients being identified as potential treatment failures at 1000cp/ml, requiring adherence counseling and further plasma-based VL testing for confirmation.

The overall testing error rate reported in our study for all specimens types acceptable at 2.5–4.6%. Although all plasma specimens reported as an error were retested, this would have implications for overall program costs in future. No plasma specimen caused inhibition (which would have resulted in an invalid result) however, the invalid rates for whole blood and DBS were high. This was most likely due to the specimen preparation buffer used in the study. We did address this on a subset of specimens (58 whole blood and 29 DBS) using a less stringent 20mM Tris-based buffer (pH8.6), and observed a decrease in the number of invalids. Further research and optimization of these protocols are required on a larger sample size to ensure feasibility for use in the field.

Overall, the Xpert VL assay was as simple to perform as the Xpert MTB/RIF (TB diagnostic) assay and training required for users already proficient in GeneXpert functionality would be minimal. A single GeneXpert instrument can perform both Xpert VL and the Xpert MTB/RIF tests concurrently, using the same instrument software with different assay definition files (ADF). Since the Xpert MTB/RIF requires 120minutes/module to complete a TB test and the Xpert VL requires 90minutes/module to complete a VL test, a site could process either four TB specimens or five HIV specimens per module in an eight hour day. Some operational factors should however be considered: color of the cartridges are the same for all Xpert assays and therefore, the need for separate specimen preparation areas; laboratory workflow and protocol needs for which specimen types take testing precedence (HIV or TB); the need for additional equipment (centrifuge for plasma testing).

Based on the Xpert VL assay performance in this study, suggested placement within the proposed South African tiered (also known as level of service facility) laboratory framework could be as follows [3]: Tier 5 could continue to provide high volume, centralized plasma-based testing in reference facilities with current platforms such as Roche and Abbott, with expansion of plasma testing to tier 4 and tier 3 using GeneXpert 48/80/16/4 instruments that are currently placed at regional and district level laboratories and where technical and implementation experience is already in place with this technology. Critical to this potential future implementation, however, is still the analysis of throughput and current GeneXpert utility if this platform is to be optimised for polyvalent testing. As previously reported [22], one technical staff member can report 93 patient results on the Abbott HIV-1 RT in a eight hour shift using one m2000sp for extraction and one m2000rt for amplification and detection. An additional 93 specimens can be extracted but not amplified in this same eight hour day. Similarly, one staff member can process 45 patient results on a Roche TaqMan 48 instrument in the same time period. This number has dramatically increased to 960 specimens per eight hour shift with the new Roche 8800 technology [23].The GeneXpert Infinity 80 (80 module system) allows for the processing of 403 samples in an eight hour period, 80 samples can be loaded, another 80 kept in the loading bay and an additional 23 on the conveyor.

As experienced in the TB Xpert MTB/RIF program, every newly placed GeneXpert instrument in the field requires verification to determine the instrument is ‘fit for purpose’ before testing and reporting of clinical results [20]. Additionally, verification is recommended when the instrument is moved and modules are replaced following instrument calibration. The same would apply to instruments performing Xpert VL testing and hence, our group explored using DBS and DPS as a Dried Viral Spot program based on the Dried Culture Spot program for TB [19]. In the absence of an Xpert-specific DBS/whole blood buffer, we chose to use water as an elution medium for the development of our Dried Viral Spot program, as this would not only cut costs of a EQA program in future but also ensure that any site can perform testing without the need to make up their own buffers or buy pre-made buffers. The DPS and DBS formats were shown to be easy to use and compatible with the Xpert VL assay and stable for up to 42 days at room temperature. Further field testing is underway, as are aspects of real-time monitoring (same as for Xpert MTB/RIF through Remote Xpert [24]).

Overall, the Xpert VL plasma assay is compatible and thus interchangeable with existing VL technologies in South Africa and lends itself well as a potential solution to extend current testing to tier 4 and 3 sites that have capacity to test plasma specimens. The Xpert provides a VL result in 90 minutes and due to the nature of the GeneXpert platform design and polyvalency (provides for testing of multiple analytes on the same platform), provides a real option to meet a range of capacity and testing needs.
